# Energy expenditure during treadmill walking with a commercial plantar-vibration shoe and internal-focus attentional instructions in healthy young women: an exploratory randomized crossover study

**DOI:** 10.1186/s13102-026-01930-3

**Published:** 2026-07-29

**Authors:** Ryota Okoba, Masaki Hasegawa

**Affiliations:** Department of Physical Therapy, Faculty of Health and Welfare, Prefectural University of Hiroshima, 1-1 Gakuen-Cho, Mihara, Hiroshima 723-0053 Japan

**Keywords:** Energy expenditure, Plantar vibration, Attentional focus, Indirect calorimetry, Treadmill walking, Randomized crossover, Exploratory study

## Abstract

**Background:**

Walking is accessible, but strategies that increase energy expenditure (EE) without additional time may be useful. We examined EE during treadmill walking with a commercial plantar-vibration shoe combined with internal-focus attentional instructions in healthy young women.

**Methods:**

Twenty-one healthy female university students completed four randomized 5-min treadmill walking trials. This analysis compared three vibration-shoe conditions: non-vibration (Vib-Off), vibration with instruction to feel it strongly (Vib-On), and vibration with instruction to attend to vibration and tactile insole protrusions (Vib+PSI). A conventional-shoe Control trial was used only to code actual trial position. Speed was constant within participants. Breath-by-breath VO2 and VCO2 were measured, and Weir-derived EE was averaged over 180–300 s. Both vibration conditions involved internal focus; vibration characteristics were not objectively measured.

**Results:**

Median EE was 2.66, 2.82, and 2.82 kcal/min in Vib-Off, Vib-On, and Vib+PSI, respectively (Friedman chi-square = 13.802, *p* = 0.001). EE was higher versus Vib-Off in Vib-On (Z = -2.850, Bonferroni-adjusted *p* = 0.013; paired Hodges-Lehmann difference 0.17 kcal/min, BCa 95% CI 0.07–0.27) and Vib+PSI (Z = -3.267, Bonferroni-adjusted *p* = 0.003; difference 0.20, BCa 95% CI 0.11–0.50). Vib+PSI did not differ from Vib-On (difference 0.02, BCa 95% CI -0.04–0.15; Bonferroni-adjusted *p* = 1.000). In the preferred mixed-effects model using actual trial position 1–4, no period term was significant and both contrasts versus Vib-Off remained significant; in a secondary compressed-period model, only Vib+PSI remained significant.

**Conclusions:**

Commercial plantar-vibration-shoe walking under internal-focus instructions was associated with small EE differences. The absolute magnitude was small, practical significance and measurement-error exceedance are uncertain, and vibration-specific effects cannot be isolated. Findings are preliminary and hypothesis-generating.

**Trial registration:**

UMIN Clinical Trials Registry, UMIN000061453. Registered 5 May 2026. Retrospectively registered.

**Supplementary Information:**

The online version contains supplementary material available at https://doi.org/10.1186/s13102-026-01930-3.

## Background

Insufficient physical activity is a global public health concern, and regular walking is widely recommended because it is feasible and acceptable for many individuals [1–3]. However, lack of time is a common barrier to increasing physical activity, and interventions that raise EE during habitual activities-without increasing session duration-could have practical value if the increase is sufficiently large and sustainable [1, 3].

Augmenting plantar sensory input is one potential approach for altering gait control and sensory-motor regulation during walking. Subthreshold or random-noise stimulation can enhance tactile perception and sensorimotor function (stochastic resonance) [4–11], and plantar stimulation, such as vibration or textured insoles, has been reported to modify gait control in various populations [12–16]. Because gait control, stability-related adjustments, and altered neuromuscular coordination can influence metabolic cost, plantar sensory manipulation may affect the energetic cost of walking at a given speed.

Beyond sensorimotor effects, attentional factors may also influence movement control during walking. Research on attentional focus suggests that explicit, conscious attention to movement or bodily sensations (internal focus) can disrupt automaticity and reduce movement efficiency compared with more automatic control [17–19]. Therefore, instructing participants to attend to plantar sensation constitutes an internal-focus manipulation. Importantly, even instructing participants to "feel the vibration strongly" may engage internal-focus processes, which limits the ability to isolate pure vibration effects from attentional effects in studies using such instructions.

Evidence regarding treadmill versus overground walking indicates that physiological and biomechanical differences can vary across environments, underscoring the importance of speed control and protocol transparency when interpreting EE outcomes [20]. In addition, estimating metabolic cost from short walking bouts can be sensitive to data-processing choices, respiratory variability, and steady-state attainment [21–24].

The same experimental sessions also generated lower-limb surface electromyography data, which were reported separately in a Japanese-language article focused on neuromuscular outcomes [25]. Because that prior report did not include respiratory-gas variables or EE outcomes, the present study specifically addresses the metabolic aspect of the intervention.

Therefore, this exploratory study examined whether walking with a commercially available plantar-vibration shoe under internal-focus attentional instructions was associated with differences in EE during treadmill walking compared with a non-vibration condition. We also explored whether adding explicit attention to tactile insole protrusions produced additional changes compared with vibration-focused instruction alone. Because both vibration conditions included internal-focus instructions and no vibration-only/no-instruction condition was included, the study was not designed to isolate the independent effects of plantar vibration from attentional focus. Given the novel commercial device, the absence of objectively characterized vibration parameters, and the absence of device- and protocol-specific measurement-error thresholds for this short walking protocol, the study was designed as exploratory and hypothesis-generating rather than confirmatory.

## Methods

### Study design and registration

This randomized, within-participant crossover study comprised four 5-min treadmill walking conditions completed in one session: a conventional athletic-shoe Control condition and three conditions using the vibration-shoe system (Vib-Off, Vib-On, and Vib+PSI). The present manuscript reports the three vibration-shoe conditions; the Control condition was excluded from the primary EE comparison but its randomized position was retained for the preferred order-effect sensitivity analysis. The full four-condition order was randomized using computer-generated sequences. Participant blinding was not feasible because vibration was perceivable; no sham or placebo condition was included. To reduce analysis bias, data processing and statistical analyses were performed using coded condition labels. Each trial consisted of 5 min of treadmill walking. All four walking conditions were completed within a single experimental session, with a 2-min standing rest interval between trials.

The present manuscript reports previously unpublished respiratory-gas and EE outcomes from the same experimental sessions and participant cohort as a prior Japanese-language article, which focused exclusively on lower-limb surface electromyography outcomes under the same three walking conditions [25]. The prior article did not report VO2, VCO2, indirect-calorimetry-derived EE, or the analyses presented here.

This trial was retrospectively registered in the UMIN Clinical Trials Registry (UMIN000061453) on 5 May 2026.

### Participants

Healthy female university students (n = 21) were recruited. Exclusion criteria were any musculoskeletal, neurological (including peripheral neuropathy), or cardiopulmonary conditions that could affect walking, as well as any self-reported contraindications or prior adverse reactions to vibration exposure. All participants provided written informed consent prior to participation. Because registration took place after participant enrollment, the trial was registered retrospectively. We acknowledge that prospective registration is recommended for randomized interventions and will prospectively register future confirmatory trials.

### Sample-size consideration

No formal a priori sample-size calculation was performed. Although effect sizes could potentially be approximated from metabolic measurement error or from previous attentional-focus studies, no directly applicable estimate was available for the present commercial plantar-vibration shoe, instruction protocol, and short-bout treadmill EE outcome. The sample size was therefore based on feasibility. To characterize the detectable effect under the present design, we conducted a post hoc sensitivity calculation. With n = 21, two-sided alpha = 0.0167 (Bonferroni adjustment for three pairwise comparisons), and 80% power, the minimum detectable standardized mean difference for a paired comparison was approximately Cohen's dz = 0.76. Accordingly, the study should be considered underpowered for detecting small effects. Separately, because device- and protocol-specific minimal detectable differences were not established, the present study could not determine whether small observed changes exceeded measurement error. For contextual purposes, a meta-analysis comparing external and internal attentional focus reported a small pooled effect on behavioral motor performance (Hedges' g = 0.264, 95% CI 0.217 to 0.310) [26]. This estimate was not directly applicable to sample-size planning for the present study because the comparison, outcome, and study designs differed: the meta-analysis examined external versus internal focus and behavioral motor-performance outcomes, whereas the present study examined combined vibration/instruction conditions and EE. Nevertheless, the estimate illustrates that attentional effects may be substantially smaller than the minimum standardized paired effect detectable with 80% power under the present design (dz approximately 0.76). Accordingly, the non-significant Vib+PSI versus Vib-On comparison should not be interpreted as evidence of equivalence or absence of an incremental effect.

### Experimental conditions

In all three analyzed conditions, participants wore the same commercially available vibration sneaker (MEGLOOP; Aoiro Fabric Co., Ltd., Aichi, Japan) with an integrated tactile insole. According to manufacturer information available from a commercial crowdfunding platform [27], the shoe incorporates magnet-based passive vibration parts embedded in the sole and does not require an external power source. In the present experiment, the vibration shoes were used in their commercially available configuration. The vibration was perceivable to participants; however, no accelerometer, vibrometer, or other device was used to objectively quantify the vibration stimulus during the experiment. Therefore, dominant frequency, acceleration magnitude, displacement amplitude, duty cycle, spatial distribution across the plantar surface, and attenuation at the shoe-foot interface during walking were not measured. No subjective ratings of perceived vibration intensity, tolerability, or exertion were collected.

In Vib-Off, vibration was disabled. In Vib-On, vibration was enabled, and participants were instructed to feel it strongly, directing their attention to plantar sensations. In Vib+PSI (vibration plus perceptual stimulus insole), vibration was enabled, and participants were instructed to consciously attend to both the vibration and the tactile protrusions of the insole. Thus, Vib+PSI represented a more explicit internal-focus instruction, but both vibration conditions involved internal focus on plantar sensations.

### Walking speed

Treadmill speed was set individually based on the average speed of two 10-m overground walks while wearing the vibration shoe. The assigned treadmill speed was kept constant across all four trials for each participant.

### Respiratory-gas measurement and EE calculation

Oxygen uptake (VO2) and carbon dioxide output (VCO2) were recorded breath-by-breath using a metabolic cart (AE-310S Aeromonitor; Minato Medical Science Co., Ltd., Osaka, Japan). At the beginning of each measurement day, the gas analyzers and flow sensor of the AE-310S were calibrated in accordance with the manufacturer's procedures, using the manufacturer-supplied reference gas and calibration syringe, respectively. The manufacturer describes the AE-310S as a breath-by-breath metabolic system that corrects gas-concentration response delay and waveform distortion and calculates VO2 and VCO2 using flow-sensor data; the flow-meter specification lists a measurement accuracy of ± 3% of the indicated value [28]. Previous comparison studies have used the AE-310S as a stationary reference system and reported intraclass correlation coefficients of 0.98 for VO2, VCO2, and VE when compared with portable metabolic systems [29, 30]. However, these studies also identified systematic between-device biases and did not establish within-device test–retest reliability or minimal detectable differences relevant to the present within-participant comparison. Therefore, they do not provide device- and protocol-specific test–retest coefficients of variation or minimal detectable differences for EE during short-duration treadmill walking using the present setup. Because device- and protocol-specific test–retest reliability for the AE-310S was unavailable, we identified external reference values from other breath-by-breath systems. A COSMED K5 study using a metabolic simulator reported minimum detectable changes of 3.8% for VO2 and 3.7% for VCO2, with technical errors no greater than 1.4% [31]. A human between-day test–retest study using the COSMED K4b2 during submaximal treadmill walking reported minimum detectable changes for gross VO2 of approximately 7–10% [32]. These estimates differed from the present study in device, outcome, testing interval, and steady-state conditions and were therefore treated as contextual benchmarks only, not as measurement-error thresholds for Weir-derived EE under the present protocol.

EE (kcal/min) was calculated using the Weir equation: EE = 3.941 × VO2 (L/min) + 1.106 × VCO2 (L/min) [33]. VO2 and VCO2 were converted from mL/min to L/min before calculation. Because breath-by-breath timestamps are not strictly equally spaced, time-weighted averages were computed.

### Representative analysis window

Each condition consisted of 5 min of walking. The representative EE for each condition was defined as the time-weighted average from 3–5 min (180–300 s), corresponding to the final 120 s. This window was selected to reduce early transient kinetics and to approximate steady state within the constraints of a short protocol [21–24]. Within-window slopes and coefficients of variation (CVs) for EE and VO2 were reported to characterize temporal stability (Table S1).

### Statistical analysis

Friedman tests and Wilcoxon signed-rank tests were performed using IBM SPSS Statistics for Windows, version 29.0 (IBM Corp., Armonk, NY, USA). Condition effects on EE were tested using Friedman’s test. When significant, two-sided asymptotic Wilcoxon signed-rank tests with continuity correction were performed for the three pairwise comparisons; zero differences were excluded from the signed-rank statistic. Bonferroni-adjusted p values were calculated as min(unadjusted p × 3, 1.000). Effect size was computed as r =|Z|/sqrt(n), where n was the number of non-zero paired differences. The same Bonferroni adjustment was applied to the three pairwise comparisons for the distance-normalized and body-mass-normalized secondary outcomes. For the primary outcome, paired Hodges-Lehmann estimates were defined as the median of the Walsh averages of paired differences. Bias-corrected and accelerated bootstrap 95% CIs were obtained using participant-level resampling with 10,000 repetitions and a fixed random seed in Python 3.13.5 with SciPy 1.17.0. As the preferred order-effect sensitivity analysis, a linear mixed-effects model was fitted using EE as the dependent variable, condition and the actual randomized trial position (1–4) as categorical fixed effects, and participant as a random intercept (Table S6A). The model was estimated by restricted maximum likelihood using the L-BFGS optimizer in statsmodels 0.14.6; Wald z tests and normal-approximation 95% CIs were reported, with Vib-Off and actual period 1 as reference categories. A secondary model renumbered the three analyzed conditions as relative periods 1–3 after excluding Control (Table S6B). These analyses were used to assess robustness rather than to replace the prespecified non-parametric primary analysis.

Protocol adherence was assessed by comparing treadmill speed during 3–5 min across conditions. We also reported distance-normalized cost (kcal/km), body-mass-normalized EE (kcal/kg/min), directional counts of individual changes (increase, decrease, or no change), and the LMM sensitivity analysis. Supplementary materials are provided as Additional file 1 (Tables S1-S9) and Additional file 2 (Figures S1-S2). Individual participant EE values are provided in Table S9.

### Use of generative artificial intelligence

During manuscript revision, ChatGPT (OpenAI) was used to assist with language refinement, document organization, and the drafting and checking of reproducibility code. It was not used to generate or alter study data. The authors independently verified all analyses, numerical results, references, and final wording and take full responsibility for the content.

## Results

### Participants, randomization sequence, and protocol adherence

Twenty-one participants completed all conditions. Participant characteristics are summarized in Table 1. Mean (± SD) age was 20.9 ± 0.8 years, height 158.5 ± 5.5 cm, body mass 50.7 ± 6.2 kg, and BMI 20.12 ± 1.53 kg/m2.

**Table 1:** Participant characteristics (*n* = 21)

Variable	Value (Mean ± SD)
Age (years)	20.9 ± 0.8
Height (cm)	158.5 ± 5.5
Body mass (kg)	50.7 ± 6.2
BMI (kg/m2)	20.12 ± 1.53

The distribution of randomized condition orders across the three analyzed conditions is provided in Table S7. After excluding Control from the sequence labels, the six possible sequences were represented as follows: Vib-Off- > Vib-On- > Vib+PSI (n = 3), Vib-Off- > Vib+PSI- > Vib-On (n = 4), Vib-On- > Vib-Off- > Vib+PSI (n = 1), Vib-On- > Vib+PSI- > Vib-Off (n = 7), Vib+PSI- > Vib-Off- > Vib-On (n = 1), and Vib+PSI- > Vib-On- > Vib-Off (n = 5).

Treadmill speed during the representative window did not differ across conditions (Table 2; median 4.30 km/h across conditions; Friedman *p* = 0.368).

**Table 2:** Treadmill walking speed by condition (km/h)

Condition	Median [Q1, Q3]	Mean ± SD
Vib-Off	4.30 [4.20, 4.30]	4.29 ± 0.20
Vib-On	4.30 [4.20, 4.30]	4.28 ± 0.19
Vib+PSI	4.30 [4.30, 4.30]	4.31 ± 0.22

Friedman *p* = 0.368. Values are presented as median [Q1, Q3] and mean ± SD

### Primary EE outcome

EE differed significantly across conditions (Table 3; Friedman chi-square = 13.802, *p* = 0.001). Median EE was 2.66 kcal/min in Vib-Off, 2.82 kcal/min in Vib-On, and 2.82 kcal/min in Vib+PSI. Post hoc tests (Table 4) showed higher EE in Vib-On than Vib-Off (Z = −2.850, *p* = 0.013, r = 0.622; paired HL difference 0.17 kcal/min, BCa 95% CI 0.07 to 0.27) and higher EE in Vib+PSI than Vib-Off (Z = −3.267, *p* = 0.003, r = 0.730; paired HL difference 0.20 kcal/min, BCa 95% CI 0.11 to 0.50). Vib+PSI did not differ from Vib-On (Z = −0.624, *p* = 1.000, r = 0.143; paired HL difference 0.02 kcal/min, BCa 95% CI −0.04 to 0.15). The paired HL differences represented approximately 6–8% relative increases compared with the Vib-Off median. Individual participant values are provided in Table S9, and the paired within-participant pattern is visualized in Figure S2 (Additional file 2).

**Table 3:** Energy expenditure (EE) by condition (kcal/min)

Condition	Median [Q1, Q3]	Mean ± SD
Vib-Off	2.66 [2.53, 2.93]	2.73 ± 0.32
Vib-On	2.82 [2.70, 3.10]	2.90 ± 0.32
Vib+PSI	2.82 [2.70, 3.09]	3.03 ± 0.63

Friedman chi-square = 13.802, *p* = 0.001. Values are presented as median [Q1, Q3] and mean ± SD

**Table 4:** Post-hoc pairwise comparisons and paired Hodges-Lehmann estimates for the primary outcome (EE, kcal/min)

Comparison	Z	n	Bonferroni-adjusted p	Effect size r	Paired HL difference [BCa 95% CI]
Vib-On vs Vib-Off	−2.850	21	0.013	0.622	0.17 [0.07, 0.27]
Vib+PSI vs Vib-Off	−3.267	20	0.003	0.730	0.20 [0.11, 0.50]
Vib+PSI vs Vib-On	−0.624	19	1.000	0.143	0.02 [−0.04, 0.15]

n indicates the number of non-zero paired differences included in the Wilcoxon signed-rank test; pairs with zero difference were excluded. Paired Hodges-Lehmann estimates are medians of Walsh averages; 95% CIs are BCa bootstrap intervals based on 10,000 participant-level resamples

### Secondary outcomes and sensitivity analyses

Distance-normalized cost (kcal/km) showed the same directional pattern of between-condition differences as the primary EE analysis (Table S3). Body-mass-normalized EE (kcal/kg/min) was higher in both Vib-On and Vib+PSI than Vib-Off (Table S4; Bonferroni-adjusted *p* = 0.0240 and *p* = 0.0011, respectively), whereas Vib+PSI did not differ from Vib-On (adjusted *p* = 1.0000). Directional counts indicated that EE increased in 17/21 participants (81.0%) for Vib-On versus Vib-Off and in 17/21 participants (81.0%) for Vib+PSI versus Vib-Off (Table S5). These counts are descriptive and were not used to define a clinical responder phenotype.

In the preferred LMM using the actual randomized trial position (1–4), no period term was statistically significant (all p > = 0.420). Relative to Vib-Off, the adjusted EE difference was 0.169 kcal/min for Vib-On (95% CI 0.001 to 0.336; *p* = 0.049) and 0.298 kcal/min for Vib+PSI (95% CI 0.130 to 0.466; *p* = 0.001) (Table S6A). In the secondary model using relative order 1–3 after excluding Control, period terms again were not significant; the Vib+PSI estimate remained significant, whereas the Vib-On estimate remained positive but was not statistically significant (Table S6B).

### Temporal stability within the analysis window

Time-course plots showed an initial rise in EE during the first minutes of each 5-min segment, followed by relative stabilization (Figure S1, Additional file 2). Within the 3–5 min window, median slopes were near zero (Table S1). However, median breath-by-breath CV values of approximately 11–13% indicated substantial within-window variability.

## Discussion

### Principal findings

This exploratory randomized, within-participant crossover study examined EE during treadmill walking with a commercial plantar-vibration shoe and internal-focus attentional instructions in healthy young women. In the primary non-parametric analysis, Vib-On and Vib+PSI were associated with higher EE than Vib-Off, with paired HL differences of 0.17 and 0.20 kcal/min, respectively. Vib+PSI did not differ from Vib-On. In the preferred LMM retaining the actual randomized trial position (1–4), no period term was significant and both Vib-On and Vib+PSI remained higher than Vib-Off. In the secondary relative-period model, the Vib+PSI contrast remained significant, whereas the Vib-On contrast was attenuated. Taken together, these findings indicate a small statistical signal under a combined sensory-attentional intervention, but they do not establish a vibration-specific or practically meaningful increase in EE.

For transparency, lower-limb surface electromyography data from the same experimental sessions have been reported previously in a Japanese-language article [25]. The present manuscript focuses on previously unpublished respiratory-gas and EE outcomes and should be interpreted as a complementary metabolic analysis of the same experimental dataset.

### Interpretation as a combined sensory-attentional intervention

Both Vib-On and Vib+PSI included instructions directing participants to focus on plantar sensations. Therefore, the present study should be interpreted as an exploratory evaluation of a commercial vibration-shoe condition used with internal-focus instructions, rather than as a test of plantar vibration alone. The observed EE differences may reflect mechanical stimulation, attentional demands, expectancy or demand characteristics, altered breathing patterns, changes in gait or muscle activity, or a combination of these factors.

This interpretation is consistent with attentional-focus literature suggesting that conscious internal focus can interfere with automatic movement control [17–19]. It is also compatible with prior plantar-stimulation studies showing that mechanical or textured plantar input can modify gait control [12–16]. A plausible, but unconfirmed, explanation is that perceivable plantar input combined with internal focus increased conscious control or altered sensorimotor regulation during walking, thereby slightly increasing energetic cost. Because the present manuscript did not include a manipulation check, kinematic analysis, or concurrent neuromuscular analysis, this mechanism remains speculative. The absence of a Vib+PSI versus Vib-On difference suggests that adding attention to tactile protrusions did not produce an identifiable incremental metabolic effect beyond the vibration-focused instruction.

### Stimulus characterization and reproducibility

The findings also need to be interpreted in relation to the uncharacterized commercial stimulus. Because the physical vibration dose delivered to the foot was not quantified, this study cannot be aligned with a specific vibration frequency, acceleration magnitude, or dose–response framework. The results therefore reflect preliminary observations during practical use of a commercially available plantar-vibration shoe combined with internal-focus attentional instructions, not evidence that a particular vibration parameter increases EE.

### Measurement precision and practical significance

The absolute magnitude of the observed EE differences was small. A paired HL difference of 0.17–0.20 kcal/min corresponds to approximately 10.2–12.0 kcal over 60 min of walking, assuming that the effect is sustained for that duration. This extrapolation is uncertain because only 5-min walking bouts were tested. Therefore, the present findings should not be interpreted as evidence of clinically or practically meaningful increases in daily EE.

The small absolute effect should also be interpreted in light of measurement uncertainty. The manufacturer reports a flow-meter accuracy of ± 3% of the indicated value for the AE-310S [28], and previous comparison studies using the AE-310S as a stationary reference system reported high agreement with portable metabolic systems [29, 30]. However, device- and protocol-specific test–retest CVs and minimal detectable differences for EE using the present AE-310S short-duration treadmill-walking protocol were not established in our laboratory and could not be identified in the literature.

For context, published benchmarks from other breath-by-breath systems span values below and above the observed group-level differences of approximately 6–8%. A simulator-based study of the COSMED K5 reported minimum detectable changes of 3.8% for VO2 and 3.7% for VCO2, reflecting predominantly technical variability [31], whereas a human between-day study using the COSMED K4b2 reported minimum detectable changes for gross VO2 of approximately 7–10% during submaximal treadmill walking [32]. The simulator-based values exclude biological variability and may underestimate uncertainty during human testing, whereas the between-day values include day-to-day biological variation and may overestimate uncertainty relevant to a within-session comparison. Because both studies also differed from the present study in device, outcome, testing interval, and steady-state conditions, these numerical comparisons are contextual only and cannot establish whether the present EE difference exceeded or fell below measurement error.

Furthermore, these published minimum detectable-change values characterize individual-level change and should not be treated as statistical significance thresholds for a group-level paired comparison. A between-condition difference smaller than an individual-level threshold can still be detected at the group level because the sampling uncertainty of the group estimate decreases with sample size. Nevertheless, we do not claim that the observed 0.17–0.20 kcal/min differences exceeded measurement error, because no device- and protocol-specific threshold was established for the AE-310S under the present protocol. The appropriate interpretation is that a small group-level difference was observed, without evidence that an individual participant would reliably increase EE under the present intervention condition.

### Period effects, carryover, and sensitivity analysis

Order effects and carryover remain important considerations because all four conditions were completed within a single session with only a 2-min standing rest interval. However, the original session included a fourth Control trial that occupied an actual randomized position. The preferred LMM therefore retained actual trial positions 1–4 rather than compressing the three analyzed conditions into periods 1–3. In this preferred model, no period term was significant, and both Vib-On and Vib+PSI remained higher than Vib-Off (Table S6A). The secondary compressed-period model produced a positive but non-significant Vib-On estimate, whereas the Vib+PSI effect remained significant (Table S6B). Thus, the Vib+PSI finding was robust to period definition, whereas the Vib-On finding was less stable and should be interpreted with greater caution.

Because sequence allocation was randomized but not perfectly balanced (Table S7), residual order or carryover effects cannot be excluded in this small sample. Nevertheless, the corrected actual-position model did not support a systematic negative period drift. Potential incomplete recovery, habituation, fatigue, or measurement drift should therefore be considered as plausible but unconfirmed sources of variation rather than as demonstrated period effects.

### Individual variability and possible mechanisms

Individual responses were heterogeneous (Table S9). Some participants showed large increases in EE, whereas others showed little change or decreases. Without objective vibration measurements, sensory-perception assessments, kinematic data, or concurrent analysis of neuromuscular responses in the present manuscript, whether this heterogeneity reflects variability in stimulus delivery, perception, physiological responsiveness, gait strategy, breathing pattern, or measurement noise remains unclear. Future studies should investigate potential moderators and mediators, including plantar sensitivity, foot biomechanics, attentional traits, perceived vibration intensity, perceived exertion, gait kinematics, and muscle activity.

Respiratory exchange ratio values were similar across conditions (median 0.861–0.883), suggesting broadly comparable exercise intensity and mixed substrate utilization across trials. Although Vib+PSI showed a slightly higher median RER, the absolute difference was small and was not a prespecified outcome; therefore, it should not be overemphasized as evidence of a distinct metabolic mechanism.

### Strengths

Despite these limitations, the study has several strengths. We used a randomized within-participant crossover design with strict control of treadmill speed, which reduced variance associated with between-participant differences. We reported effect sizes and HL differences with confidence intervals rather than relying solely on p values, and we conducted sensitivity analyses with an LMM including trial period as a covariate. Individual participant data for the primary EE outcome are provided in the supplementary material (Table S9), allowing readers to inspect response heterogeneity. The transparent reporting of key limitations, including attentional confounding, absence of objective vibration characterization, measurement uncertainty, and period effects, helps define the methodological requirements for future confirmatory trials.

### Summary of interpretation

Overall, this exploratory study suggests that EE during controlled treadmill walking may differ slightly when healthy young women walk with a commercial plantar-vibration shoe under internal-focus attentional instructions. However, critical limitations-including inability to isolate vibration-specific effects, unknown vibration characteristics, lack of blinding or placebo control, possible period-related carryover, unbalanced sequence distribution, heterogeneous individual responses, and small absolute effect sizes relative to measurement uncertainty-preclude firm conclusions. The findings should therefore be regarded as preliminary and hypothesis-generating.

### Limitations

Objective vibration characteristics were not measured, and detailed physical parameters were not available from the manufacturer. The only publicly available product information came from a commercial crowdfunding platform [27], which is insufficient for scientific reproducibility. Because frequency, acceleration magnitude, displacement amplitude, duty cycle, spatial distribution, and shoe-foot transmission were unknown, the study cannot be replicated as a precisely dosed vibration intervention and cannot support vibration-specific mechanistic conclusions. Table S8 provides a template for vibration parameters that should be objectively quantified in future work.

The intervention conditions did not allow vibration effects to be separated from attentional effects. Both vibration conditions involved instructions to focus on plantar sensations, and no vibration-only/no-instruction, attention-neutral, sham vibration, or placebo condition was included. Participants could not be blinded to the presence or absence of vibration, raising the possibility of expectancy effects, demand characteristics, altered breathing patterns, or changes in movement control unrelated to vibration dose. In addition, no manipulation check was collected to determine whether participants adopted the intended attentional strategies or whether Vib-On and Vib+PSI produced different cognitive states.

Measurement precision also limits interpretation. Although the 3–5 min analysis window was selected to reduce transient kinetics, true steady state cannot be guaranteed with 5-min walking bouts. Median within-window CVs of approximately 11–13% indicate substantial breath-by-breath variability, and device- and protocol-specific test–retest CVs and minimal detectable differences for EE using the present AE-310S treadmill-walking protocol were not established. External reference values from K5 and K4b2 studies provide only order-of-magnitude context and cannot be applied as protocol-specific thresholds because the device, outcome, testing interval, and steady-state conditions differed [31, 32]. Therefore, we cannot determine whether the observed 0.17–0.20 kcal/min differences exceeded measurement error.

Potential period effects and carryover represent another important limitation. The original session included four randomized conditions, and the preferred LMM retained the actual trial positions 1–4. No actual-position term was significant, but the sequence distribution was not perfectly balanced and only a 2-min standing rest interval separated trials. Residual post-walking metabolic elevation, habituation, fatigue, or measurement drift therefore cannot be excluded. A secondary model that compressed the analyzed conditions into relative periods 1–3 attenuated the Vib-On estimate, indicating that the Vib-On result is less robust to period definition than the Vib+PSI result.

The sample and protocol limit generalizability. Participants were healthy young women only, and menstrual cycle phase and habitual physical activity were not controlled. Treadmill speed was determined from short 10-m overground walks that included acceleration and deceleration phases, and treadmill walking differs biomechanically and potentially metabolically from overground walking [20]. Consequently, the findings may not generalize to men, older adults, clinical populations, longer walking durations, or free-living walking.

Individual responses were heterogeneous, with some participants showing large increases in EE and others showing decreases (Table S9). Because no a priori rule for excluding extreme individual responses was specified and no documented measurement artifact was identified, no post hoc outlier exclusion was performed. Future studies should prespecify rules for handling extreme values and should include measures of stimulus delivery, perception, movement strategy, and respiratory response to clarify sources of heterogeneity.

The practical significance of the observed differences is uncertain. The primary paired HL differences of 0.17–0.20 kcal/min correspond to only approximately 10.2–12.0 kcal over 60 min of walking if the effect is sustained, which was not tested. The results should therefore not be interpreted as evidence that this intervention meaningfully increases daily EE or supports weight-management applications.

Future studies should objectively measure vibration parameters using accelerometry or vibrometry during loaded walking; include vibration-only, sham, placebo, and attention-neutral control conditions; incorporate manipulation checks and perceived-intensity ratings; use longer protocols with sufficient washout intervals; ensure balanced randomization sequences; prospectively register randomized interventions; and test broader participant samples in overground or free-living contexts.

## Conclusions

In this exploratory study, walking with a commercial plantar-vibration shoe under internal-focus attentional instructions was associated with small statistical differences in EE during controlled treadmill walking in healthy young women. However, these differences were small in absolute magnitude, their practical significance is uncertain, and whether they exceeded measurement error remains unclear. Additional instruction to attend to tactile insole protrusions did not further increase EE beyond the vibration-focused instruction. The Vib+PSI contrast was robust to alternative definitions of trial period, whereas the Vib-On estimate was attenuated in the secondary compressed-period model. Because of attentional confounding, lack of blinding or placebo control, unknown vibration characteristics, potential carryover, unbalanced sequence distribution, heterogeneous responses, and the absence of device- and protocol-specific minimal detectable differences, the findings should be interpreted as preliminary and hypothesis-generating rather than as evidence of practically meaningful or vibration-specific increases in EE. In particular, the observed group-level differences do not imply that an individual participant would reliably increase EE when using this shoe under the present attentional-instruction protocol.

## Supplementary Information

Additional file 1. Supplementary tables (Tables S1-S9). Includes steady-state verification indices, protocol adherence, secondary outcomes, directional counts of individual changes, linear mixed-effects model results, sequence distribution, a template for reporting vibration characteristics, and individual participant EE values.

Additional file 2. Supplementary figures (Figures S1-S2). Includes the EE time-course plot and the paired individual participant EE plot.

## Data Availability

The participant-level dataset supporting the primary energy-expenditure outcome is included within the article and its Additional file 1 (Table S9). The breath-by-breath source data and datasets supporting the exploratory secondary analyses are not publicly available. Requests for access to de-identified data may be directed to the corresponding author and will be considered subject to institutional ethics and data-use requirements.

## Abbreviations

EE: Energy expenditure
VO2: Oxygen uptake
VCO2: Carbon dioxide output
RER: Respiratory exchange ratio
CV: Coefficient of variation
HL: Hodges-Lehmann
LMM: Linear mixed-effects model
BMI: Body mass index
PSI: Perceptual stimulus insole
