# Supplementary material for: Energy expenditure during treadmill walking with a commercial plantar-vibration shoe and internal-focus attentional instructions in healthy young women: an exploratory randomized crossover study
Source: BMC Sports Sci Med Rehabil. 2026 Jul 29;18:560. doi: 10.1186/s13102-026-01930-3 (PMC13737641; doi:10.1186/s13102-026-01930-3)
Supplement: Supplementary file 1 [file 13102_2026_1930_MOESM1_ESM.docx]

**Additional file 1: Supplementary tables (Tables S1-S9).**

**Table S1. Steady-state verification indices within the representative window (final 120 s of each 5-min walking segment).**

| Condition | EE slope (kcal/min per min) | EE CV (%) | VO2 slope (L/min per min) | VO2 CV (%) | RER (mean) |
| --- | --- | --- | --- | --- | --- |
| Vib-Off | 0.029 [-0.050, 0.073] | 12.965 [8.274, 15.926] | 0.005 [-0.013, 0.012] | 12.908 [8.351, 15.887] | 0.861 [0.849, 0.910] |
| Vib-On | -0.034 [-0.089, 0.019] | 11.026 [9.260, 16.829] | -0.009 [-0.021, 0.001] | 10.930 [9.022, 16.623] | 0.872 [0.858, 0.912] |
| Vib+PSI | -0.030 [-0.074, 0.009] | 10.977 [7.867, 15.781] | -0.005 [-0.014, -0.001] | 10.853 [7.912, 15.695] | 0.883 [0.855, 0.904] |

*Values are median [Q1, Q3]. Slopes were estimated by linear regression within the window; CV = coefficient of variation.*

**Table S2. Treadmill speed during the representative window (protocol adherence check).**

| Condition | Speed (km/h), median [Q1, Q3] |
| --- | --- |
| Vib-Off | 4.3 [4.2, 4.3] |
| Vib-On | 4.3 [4.2, 4.3] |
| Vib+PSI | 4.3 [4.3, 4.3] |

*Friedman χ²=2.00, p=0.368.*

**Table S3. Energy cost per distance (kcal/km): descriptive statistics and pairwise comparisons.**

| Condition | Energy cost (kcal/km), median [Q1, Q3] |
| --- | --- |
| Vib-Off | 37.58 [35.62, 41.15] |
| Vib-On | 39.40 [37.73, 42.36] |
| Vib+PSI | 38.79 [37.58, 42.62] |

**Table S3 (continued). Pairwise comparisons for energy cost per distance (kcal/km).**

| Comparison | HL diff (kcal/km) | 95% CI | Bonferroni-adjusted Wilcoxon p | Effect size r |
| --- | --- | --- | --- | --- |
| Vib-On vs Vib-Off | 1.65 | [0.52, 3.66] | 0.0098 | 0.642 |
| Vib+PSI vs Vib-Off | 1.89 | [0.97, 3.08] | 0.0005 | 0.823 |
| Vib+PSI vs Vib-On | 0.21 | [-0.72, 0.81] | 1.0000 | 0.037 |

**Table S4. Body mass-normalized EE (kcal/kg/min): descriptive statistics and pairwise comparisons.**

| Condition | EE (kcal/kg/min), median [Q1, Q3] |
| --- | --- |
| Vib-Off | 0.0529 [0.0505, 0.0579] |
| Vib-On | 0.0561 [0.0533, 0.0602] |
| Vib+PSI | 0.0566 [0.0540, 0.0640] |

**Table S4 (continued). Pairwise comparisons for body mass-normalized EE (kcal/kg/min).**

| Comparison | HL diff (kcal/kg/min) | 95% CI | Bonferroni-adjusted Wilcoxon p | Effect size r |
| --- | --- | --- | --- | --- |
| Vib-On vs Vib-Off | 0.0020 | [0.0008, 0.0049] | 0.0240 | 0.579 |
| Vib+PSI vs Vib-Off | 0.0022 | [0.0013, 0.0051] | 0.0011 | 0.780 |
| Vib+PSI vs Vib-On | 0.0003 | [-0.0011, 0.0011] | 1.0000 | 0.045 |

Wilcoxon p values were Bonferroni-adjusted for three pairwise comparisons; adjusted p < 0.05 was considered statistically significant.

**Table S5. Directional counts of individual changes in energy expenditure.**

| Comparison | Increase / decrease / no change (n) | Increase (%) |
| --- | --- | --- |
| Vib-On - Vib-Off | 17 / 4 / 0 | 81.0 |
| Vib+PSI - Vib-Off | 17 / 3 / 1 | 81.0 |
| Vib+PSI - Vib-On | 11 / 8 / 2 | 52.4 |

Increase indicates EE is higher in the left-hand condition of the comparison.

**Table S6A. Linear mixed-effects model for EE using actual randomized trial position (1-4).**

*Model: EE ~ condition + actual randomized trial position + participant-specific random intercept. Reference categories were Vib-Off and actual period 1. Estimation used REML with the L-BFGS optimizer; Wald z tests and normal-approximation 95% CIs are shown. The original session included a fourth Control trial; retaining actual positions 1-4 avoids compressing the trial timeline.*

| **Term** | **Estimate (kcal/min)** | **SE** | **z** | **95% CI** | **p-value** |
| --- | --- | --- | --- | --- | --- |
| Intercept | 2.704 | 0.125 | 21.565 | [2.459, 2.950] | <0.001 |
| Vib-On vs Vib-Off | 0.169 | 0.086 | 1.966 | [0.001, 0.336] | 0.049 |
| Vib+PSI vs Vib-Off | 0.298 | 0.086 | 3.479 | [0.130, 0.466] | 0.001 |
| Actual period 2 vs 1 | 0.041 | 0.112 | 0.363 | [-0.180, 0.261] | 0.717 |
| Actual period 3 vs 1 | 0.080 | 0.099 | 0.807 | [-0.115, 0.275] | 0.420 |
| Actual period 4 vs 1 | -0.024 | 0.111 | -0.214 | [-0.241, 0.194] | 0.831 |

**Table S6B. Secondary linear mixed-effects model using relative order 1-3 after excluding Control.**

*Model settings were otherwise identical to Table S6A. This compressed-period model is presented as a secondary sensitivity analysis.*

| **Term** | **Estimate (kcal/min)** | **SE** | **z** | **95% CI** | **p-value** |
| --- | --- | --- | --- | --- | --- |
| Intercept | 2.740 | 0.111 | 24.625 | [2.522, 2.958] | <0.001 |
| Vib-On vs Vib-Off | 0.134 | 0.087 | 1.543 | [-0.036, 0.305] | 0.123 |
| Vib+PSI vs Vib-Off | 0.249 | 0.090 | 2.755 | [0.072, 0.426] | 0.006 |
| Relative period 2 vs 1 | 0.095 | 0.085 | 1.115 | [-0.072, 0.262] | 0.265 |
| Relative period 3 vs 1 | -0.031 | 0.085 | -0.366 | [-0.197, 0.135] | 0.715 |

**Table S7. Distribution of randomized condition orders (sequence counts).**

| Sequence (1st→2nd→3rd) | n |
| --- | --- |
| Vib-Off→Vib-On→Vib+PSI | 3 |
| Vib-Off→Vib+PSI→Vib-On | 4 |
| Vib-On→Vib-Off→Vib+PSI | 1 |
| Vib-On→Vib+PSI→Vib-Off | 7 |
| Vib+PSI→Vib-Off→Vib-On | 1 |
| Vib+PSI→Vib-On→Vib-Off | 5 |

**Table S8. Template for reporting vibration and tactile-insole characteristics (to support reproducibility).**

| Item | Definition / method | Value |
| --- | --- | --- |
| Product name | Manufacturer information | MEGLOOP vibrating sneakers (commercial product) [Ref. 27] |
| Manufacturer | Manufacturer information | Aoiro Fabric Co., Ltd. (Aichi, Japan) [Ref. 27] |
| Vibration generation mechanism | Manufacturer information | Magnet-based, passive vibration parts embedded in the sole; no battery or external power source required [Ref. 27] |
| Vibration actuator location | e.g., heel/forefoot/whole sole | Shoe sole (magnetic vibration parts); precise spatial distribution not objectively quantified in the present study [Ref. 27] |
| Vibration occurrence/perception | Manufacturer information | Perceptible vibration is generated during walking and may be perceived at foot contact and/or toe-off depending on the user [Ref. 27] |
| Manufacturer guidance on perception | Manufacturer information | Some users may not perceive vibration; the manufacturer suggests confirming vibration by lightly tapping the shoe and notes that walking with a whole-foot contact may enhance perception [Ref. 27] |
| Dominant frequency (Hz) | measured with a tri-axial accelerometer | Not measured in the present study |
| RMS acceleration (m/s^2) | measured | Not measured in the present study |
| Peak-to-peak acceleration (m/s^2) | measured | Not measured in the present study |
| Duty cycle | continuous / intermittent (% on) | Not measured in the present study |
| Measurement setup | sensor model, sampling rate, attachment site | Not measured in the present study |
| Manufacturer-provided insole feature | Manufacturer information | Original insole designed to stimulate four plantar points (acupressure) [Ref. 27] |
| Tactile insole protrusion geometry | diameter/height/pitch/material (if available) | The manufacturer reports an original insole designed to stimulate four plantar points, but detailed protrusion geometry is not publicly specified. PSI protrusion geometry: Not available for reporting. [Ref. 27] |

**Table S9. Individual participant energy expenditure values by condition (kcal/min; de-identified).**

| Participant ID | Vib-Off | Vib-On | Vib+PSI |
| --- | --- | --- | --- |
| 1 | 2.84 | 2.88 | 2.84 |
| 2 | 2.30 | 2.70 | 2.77 |
| 3 | 2.63 | 2.74 | 2.75 |
| 4 | 2.76 | 2.67 | 2.70 |
| 5 | 2.98 | 2.82 | 2.78 |
| 6 | 2.51 | 2.83 | 2.76 |
| 7 | 2.45 | 2.48 | 2.48 |
| 8 | 2.65 | 2.76 | 2.83 |
| 9 | 2.13 | 2.39 | 2.36 |
| 10 | 2.57 | 2.59 | 2.70 |
| 11 | 2.66 | 3.32 | 2.94 |
| 12 | 2.53 | 2.74 | 2.66 |
| 13 | 3.39 | 3.66 | 4.96 |
| 14 | 2.56 | 3.03 | 3.68 |
| 15 | 3.33 | 3.10 | 4.39 |
| 16 | 2.49 | 2.82 | 2.82 |
| 17 | 2.93 | 3.35 | 3.17 |
| 18 | 2.97 | 3.02 | 3.09 |
| 19 | 2.66 | 2.59 | 2.60 |
| 20 | 2.82 | 3.13 | 2.84 |
| 21 | 3.19 | 3.34 | 3.56 |
