# Supplementary material for: Energy expenditure during treadmill walking with a commercial plantar-vibration shoe and internal-focus attentional instructions in healthy young women: an exploratory randomized crossover study
Source: BMC Sports Sci Med Rehabil. 2026 Jul 29;18:560. doi: 10.1186/s13102-026-01930-3 (PMC13737641; doi:10.1186/s13102-026-01930-3)
Supplement: Supplementary file 2 [file 13102_2026_1930_MOESM2_ESM.docx]

**Additional file 2. Supplementary Figures**

**Figure S1. Time-course of energy expenditure (EE) during the 5-min treadmill walking trials.**

Lines indicate the across-participant median at 1-Hz resolution, and shaded bands indicate the interquartile range (IQR). Panels show Vib-Off, Vib-On, and Vib+PSI. The shaded background denotes the primary analysis window (180–300 s).

**Figure S2. Individual participant energy expenditure (EE) by condition (paired dot plot).**

Each point represents one participant, with lines connecting within-participant values across conditions.
